# Reasons for treatment changes in adults with attention-deficit/hyperactivity disorder: a chart review study

**DOI:** 10.1186/s12888-022-04016-9

**Published:** 2022-06-03

**Authors:** Jeff Schein, Ann Childress, Martin Cloutier, Urvi Desai, Andi Chin, Mark Simes, Annie Guerin, Julie Adams

**Affiliations:** 1 Otsuka Pharmaceutical Development & Commercialization, Inc., 508 Carnegie Center, Princeton, NJ 08540 USA; 2 Center for Psychiatry and Behavioral Medicine, 7351 Prairie Falcon Rd STE 160, Las Vegas, NV 89128 USA; 3 Analysis Group, Inc., 1190 avenue des Canadiens-de-Montréal, Tour Deloitte, Suite 1500, Montréal, QC H3B 0G7 Canada; 4 Analysis Group, Inc., 111 Huntington Avenue, 14th floor, MA 02199 Boston, USA; 5 Analysis Group, Inc., 151 W 42nd St 23rd floor, New York, NY 10036 USA

**Keywords:** ADHD, Adult, Chart review, Treatment changes, Discontinuation, Complications

## Abstract

**Background:**

Adults with attention-deficit hyperactivity disorder (ADHD) often cycle through multiple treatments for reasons that are not well documented. This study analyzed the reasons underlying treatment changes among adults treated for ADHD in a real-world setting.

**Methods:**

Data were collected via an online reporting form completed by eligible physicians between October and November 2020. Data for adult patients in the United States who were diagnosed with ADHD and initiated a treatment regimen within 1 to 5 years of chart abstraction were obtained. Reason for a treatment change was described for a randomly selected regimen episode, which spanned from treatment initiation until the earliest among treatment add-on/switch or discontinuation, death, or date of chart abstraction. The overall rate of ADHD/treatment-related complications were also described. Physician satisfaction with current treatment options for adult ADHD and opinions on areas for improvement were assessed.

**Results:**

Data on 320 patients were reported by 152 physicians specializing in psychiatry (40.1%), pediatrics (25.0%), family medicine (21.7%), and internal medicine (13.2%). Patients had a mean age of 29.3 years; most were diagnosed with ADHD as adults (57.5%) and within the previous 5 years (56.5%). Selected treatment regimens included stimulants (79.1%), nonstimulants (14.7%), and combination therapy (5.6%) for an average duration of 1.9 years. Among patients with treatment discontinuation (*N *= 59), the most common reasons for discontinuation were suboptimal symptom management (55.9%), occurrence of ADHD/treatment-related complications (25.4%), and patient attitude/dislike of medication (25.4%). The main reasons for other key treatment changes were inadequate/suboptimal management of symptoms and cost considerations. Over 40% of patients had ≥ 1 documented ADHD/treatment-related complication, irrespective of whether they led to a treatment change. One in 5 physicians (19.8%) were very dissatisfied, moderately dissatisfied, or neither satisfied nor dissatisfied with current treatment options for ADHD in adults; the top 3 suggested improvements were lower risk of abuse (71.7%), longer effect duration (65.1%), and fewer ADHD/treatment-related complications (61.2%).

**Conclusions:**

The top reasons for treatment changes among adults with ADHD are lack of efficacy and ADHD/treatment-related complications, highlighting the importance of developing more effective and safer treatments to alleviate the burden of ADHD.

## Background

Attention-deficit hyperactivity disorder (ADHD) is a neuropsychiatric condition characterized by hyperactivity, inattention, and impulsivity that may affect patients throughout the lifespan [1]. ADHD has historically been studied as a disorder in children and adolescents but is increasingly recognized as affecting adults [2]. The prevalence of ADHD among adults in the United States (US) is estimated at 4.4% [3]. It has been reported that ADHD symptoms persist into adulthood in 35%–78% of childhood cases [3, 4].

ADHD in adults is associated with a wide range of psychosocial and occupational problems that may diminish patients’ quality of life [5, 6]. For instance, these individuals may exhibit deficits in executive function that affect academic, career, and relationship success; they may also be more likely to engage in high-risk behaviors (e.g., substance abuse, criminal activity, reckless behavior/accidents) [7]. Additionally, up to 80% of adults with ADHD have at least one coexisting psychiatric disorder, including mood and anxiety disorders [8–11], as well as medical comorbidities, such as obesity, sleep disorders, asthma, and migraines [12–14]. Thus, ADHD in adults constitutes a significant economic [15, 16] and societal [17, 18] burden.

Several treatment strategies for adults with ADHD have been shown to be associated with symptom improvement [7] and reduced rates of traffic accidents [19], substance abuse [20], suicidal behavior [12], and criminal convictions [21]. Current treatment options for ADHD include pharmacologic (stimulants [e.g., amphetamines and methylphenidates]) and nonstimulants [e.g., atomoxetine]) and non-pharmacologic (e.g., cognitive behavioral therapy) interventions, used either alone or in combination [7, 22]. Despite available treatment options, adherence is generally low [23, 24] and treatment discontinuations are frequent [25, 26] among adults with ADHD, suggesting unmet treatment needs in this population. Unfortunately, most existing studies in ADHD focused on children and adolescents and/or examined specific treatment patterns, such as discontinuation for a specific treatment, with few addressing issues in adults across all treatment options and considering other modifications to treatment regimens [27–29].

Recently, a large claims-based study comprehensively evaluated various types of treatment patterns (including treatment discontinuation, switch, augmentation, and drop) among adults with ADHD treated with different regimens in the US. The study found that more than half of the patients experienced a change in pharmacologic treatment within 12 months and that more frequent treatment changes were associated with higher healthcare costs [26]. While this observation underlines the potential substantial unmet needs in adult patients with ADHD, a limitation of claims-based studies is that the reasons underlying treatment changes could not be captured due to the lack of sufficient clinical information. Clarifying the reasons for treatment changes is critical for clinicians and stakeholders to comprehend the unmet treatment needs of adults with ADHD and identify potential avenues to improve patient care. To address this important knowledge gap, the current chart review study was conducted to understand the reasons underlying treatment changes among adults treated for ADHD in a real-world setting through review of medical records. Additionally, the overall rates of ADHD/treatment-related complications among patients, irrespective of whether they led to a treatment change, as well as physician satisfaction with current therapeutic options for adult ADHD and their suggestions on areas for improvement in the treatment landscape were also assessed.

## Methods

### Study design and data source

This descriptive retrospective study analyzed anonymized data obtained through online medical chart abstraction from October to November 2020. Physicians in the US who treat patients with ADHD were recruited among a panel of healthcare providers maintained by M3 Global Research, a full licensee of the American Medical Association master file. Eligible physicians (defined below) were invited to participate in the study and complete an online survey; participating physicians were asked to provide clinical information on 1 to 3 patients with ADHD via an electronic case report form designed specifically for this study. Patient-level information was collected via the electronic case report form completed by treating physicians; and physician-level information was collected through the online survey. Collected data did not include any patient-identifying information. This retrospective study was approved by the Western Institutional Review Board–Copernicus Group Institutional Review Board (Puyallup, WA).

Physicians meeting the following eligibility criteria were included in the study: 1) practice medicine in the US; 2) responsible for treatment decisions and follow-up of at least one eligible adult patient with ADHD; and 3) have access to detailed patient medical charts.

Eligibility criteria for patients were as follows: 1) initiated a new pharmacologic treatment for ADHD after the date of diagnosis, and between 1 and 5 years prior to the date of chart abstraction; 2) aged between 18 and 64 years; 3) diagnosed with ADHD; and 4) had at least one recorded medical visit with the participating physician in the 12 months prior to the date of chart abstraction.

Physicians were asked to compile a list of eligible patients along with their treatment regimens for ADHD. To reduce potential selection bias, physicians were asked to enter information for all ADHD treatments received in the previous 1 to 5 years (the eligible study period) for an individual patient whose last name began with a letter randomly generated by the survey. From among the available treatment regimens entered for each eligible patient, the survey randomly selected one ADHD treatment regimen. Patient characteristics were assessed as of the date of initiation of this regimen.

### Study measures

Physician characteristics including demographics (e.g., age, gender) and information on their practice (e.g., practice size, region) are summarized. Patient characteristics including demographic (e.g., age, gender) and clinical (e.g., type of ADHD, severity of ADHD, treatment history) variables are also summarized. To determine the reason for a treatment change, data were collected for a selected treatment regimen for each patient. The duration of the selected treatment episode spanned from the initiation of the treatment until the earliest among the following key treatment changes: treatment add-on or switch (defined as switching ≥ 1 agent in combination therapy) or discontinuation; death; or the date of chart abstraction. A change of formulation of the same agent was considered as a change of treatment. Changes related to the selected treatment episode that were analyzed included reasons for the change, treatment duration, and ADHD/treatment-related complications that contributed to the end of the treatment episode. Additional regimen modifications including treatment interruption and dose increase or decrease that occurred during but did not constitute the end of the selected treatment episode were also collected. ADHD/treatment-related complications that occurred during the selected regimen, regardless of whether they contributed to a treatment change, were also described. Physician strategies to manage common complications as well as their treatment preferences, including level of satisfaction with current ADHD therapeutic options for adults and opinions regarding potential areas for improvement in available treatments, were also described.

Means, medians, interquartile ranges, and standard deviations are reported for continuous variables. Frequency counts and percentages were reported for categorical variables. All statistical analyses were performed using SAS Enterprise Guide 7.1 and R version 3.6.3.

## Results

### Physician characteristics

The practice characteristics of the 152 physicians who participated in the study are shown in Table 1. Psychiatry was the most common specialty (40.1%), followed by pediatrics (25.0%), family medicine (21.7%), and internal medicine (13.2%); and 77.6% of physicians were in private/community practice. More than half of physicians were practicing in suburban areas (53.9%) while fewer were practicing in urban (36.2%) and rural (9.9%) areas.

**Table 1:** Physician practice characteristics

	***N* = 152**
**Physician specialty (%)**	
Psychiatry	40.1%
Pediatrics	25.0%
Family medicine	21.7%
Internal medicine	13.2%
**Practice type (%)**	
Private/community practice	77.6%
Institutional academic	13.2%
Institutional non-academic	9.2%
**Size of practice (%)**	
Individual	17.1%
2–9 physicians	48.0%
≥ 10 physicians	34.9%
**Region of practice (%)**	
Northeast	24.3%
Midwest	25.0%
South	32.2%
West	18.4%
**Setting of practice (%)**	
Suburban	53.9%
Urban	36.2%
Rural	9.9%
**Types of healthcare coverage accepted (%)**	
Commercial/private insurance	95.4%
Medicaid	77.0%
Medicare	67.8%
Military insurance (VA or active military)	54.6%
Other	5.3%
**Number of adult patients with ADHD treated on an annual basis (%)**	
1–25	26.3%
26–50	17.8%
51–75	11.8%
76–100	17.1%
≥ 101	27.0%

*ADHD* Attention-deficit/hyperactivity disorder, *VA* Veteran Affairs

### Patient characteristics

A total of 320 patient charts were contributed by participating physicians. The patients’ demographic and clinical characteristics are shown in Table 2. The mean age was 29.3 years and 65.0% of patients were male; more than half of the patients were diagnosed with ADHD as adults (57.5%) and had received the diagnosis within the previous 5 years (56.5%). The ADHD subtypes represented in the sample were inattentive (50.0%), combined (42.2%), hyperactive (7.2%), and unknown (0.6%). Most patients had moderate disease severity (75.6%), with smaller proportions having mild (11.3%) or severe (10.9%) disease.

**Table 2:** Patient demographic and clinical characteristics

	***N* = 320**
***Demographics at start of selected treatment episode***^a^	
**Age**	
Mean ± SD	29.3 ± 10.7
Median	26.9
IQR	(19.3, 36.9)
**Gender (%)**	
Male	65.0%
Female	34.7%
Non-binary	0.3%
**Race (%)**	
American Indian or Alaska Native	1.3%
Asian	8.4%
Black or African American	11.3%
Native Hawaiian or Other Pacific Islander	0.0%
White or Caucasian	76.9%
Other	1.6%
Unknown	0.6%
**Ethnicity (%)**	
Hispanic or Latino	5.9%
Not Hispanic or Latino	91.3%
Unknown	2.8%
**Region of residence (%)**	
Midwest	27.8%
Northeast	22.2%
South	31.6%
West	18.4%
**Type of healthcare coverage (%)**	
Commercial/private insurance	76.9%
Medicare	3.1%
Medicaid	15.3%
Military insurance (VA or active military)	2.8%
Other	0.0%
No insurance	1.3%
Unknown	2.8%
**Duration of treatment history available (%)**	
0–1 years	12.2%
2–3 years	39.1%
4–5 years	22.5%
≥ 5 years	26.3%
***Clinical disease characteristics***	
**Type of ADHD (%)**	
Inattentive	50.0%
Combined presentation	42.2%
Hyperactive	7.2%
Unknown	0.6%
** Age at first diagnosis (%)**	
Child	20.3%
Adolescent	22.2%
Adult	57.5%
**Time from first diagnosis to treatment initiation (%)**	
Less than 1 year	12.5%
1–5 years	44.1%
5–10 years	17.8%
10–15 years	10.9%
≥ 15 years	14.7%
**Overall disease severity**^b^ **at time of treatment initiation (%)**	
Mild	11.3%
Moderate	75.6%
Severe	10.9%
Unknown	2.2%
**Most frequent comorbidities associated with ADHD (%)**	
Anxiety	31.6%
Depression	14.1%
Insomnia/sleep disturbances	10.9%
Emotional impulsivity/lability/dysregulation	10.6%
Learning disability	5.3%
**Most frequent other comorbidities (%)**	
Obesity	6.6%
Diabetes without chronic complications	2.5%
Liver disease, mild	1.6%
Chronic pulmonary disease	1.3%
Peptic ulcer disease	1.3%

*ADHD* Attention-deficit/hyperactivity Disorder, *AE* Adverse Event, *SD* Standard Deviation, *IQR* Interquartile Range, *VA* Veteran Affairs

^a^ All patients were alive as of the date of chart abstraction

^b^ Disease severity was determined by clinical judgement in 45% of physicians, and the remaining physicians reported using ADHD rating scales, including the Adult ADHD Clinical Diagnostic Scale (ACDS), Adult ADHD Self-Report Scale (ASRS), ADHD Rating Scale IV and/or 5 (ADHD-RS-IV and/or ADHD-RS-5), Adult ADHD Investigator Symptom Rating Scale (AISRS)

Patients were treated with pharmacologic and nonpharmacologic approaches. As adults, their treatment history included an average of 2.2 individual agents, which were in most cases stimulants (95.3%) although nonstimulants were also used (31.6%). Nonpharmacologic interventions included psychotherapy (40.3%), training in organizational skills (26.6%), and academic skills (19.1%). For 136 patients, adolescent/child ADHD treatment history was also available: 58.1% received stimulants and 16.9% received nonstimulants before adulthood, while school-based interventions (31.6%), academic skills training (24.3%), and psychotherapy (18.4%) were used as adjunctive treatments.

### Characteristics of the selected treatment regimen

Stimulants were the selected treatment in 79.1% of patients; 14.7% were treated with nonstimulants; and 5.6% received combination therapy. The average duration of the selected treatment episode was 1.9 years.

### Key reasons for treatment changes and modifications

**Fig. 1:**
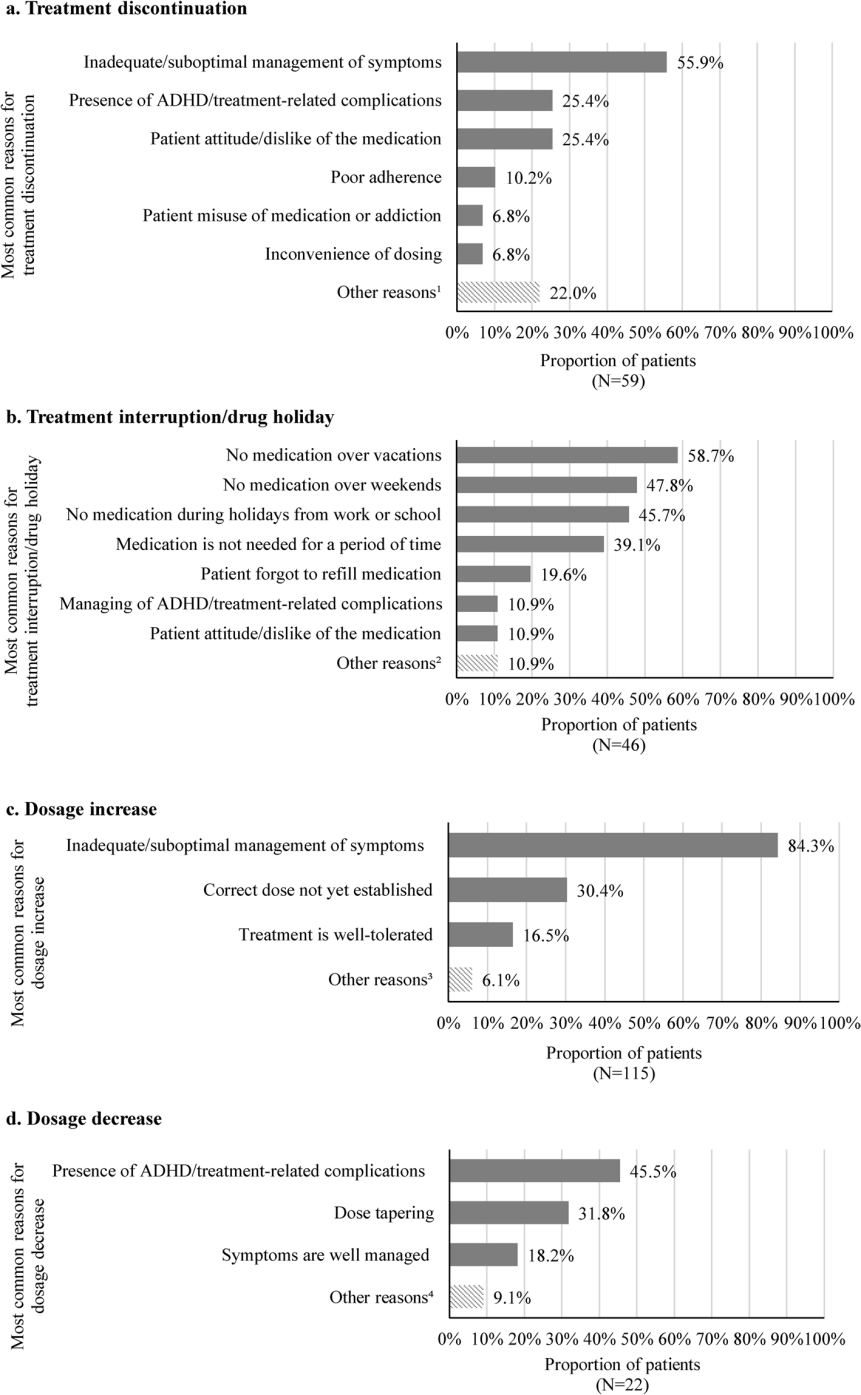
Most common reasons for changes in treatment pattern. **a** Treatment discontinuation. **b** Treatment interruption/drug holiday. **c** Dosage increase. **d** Dosage decrease. Notes: [1] Other reasons for treatment discontinuation included cost considerations, social stigma associated with ADHD medication, treatment monitoring becoming burdensome, treatment no longer being appropriate, member of patient’s household misuse of medication or addiction, and other patient-driven and physician-driven reasons. [2] Other reasons for treatment interruption/drug holiday included social stigma associated with ADHD medication, testing treatment efficacy or if treatment was still needed, and other patient-driven reason. [3] Other reasons for dosage increase included change in insurance coverage, leveraging the treatment’s side effects (e.g., weight loss in obese patient), weight gain (e.g., for dose dependent treatment), and other patient-driven and physician-driven reasons. [4] Other reasons for dosage decrease included change in insurance coverage and other patient-driven and physician-driven reasons

Among patients with treatment discontinuation (*N* = 59), the most common reasons were suboptimal symptom management (55.9%), occurrence of ADHD/treatment-related complications (25.4%), patient attitude/dislike of medication (25.4%), poor adherence (10.2%), patients misuse of medications or addiction (6.8%), and inconvenient of dosing (6.8%; Fig. 1). The most common complications leading to discontinuation were anxiety/panic attacks (13.3%) and hypertension (13.3%). The main reasons for other key treatment changes (including treatment add-on/switch) were inadequate/suboptimal management of symptoms and cost considerations.

Additionally, some patients experienced treatment modifications such as interruption and dose modification that did not lead to treatment changes. Among patients with treatment interruption/drug holiday (*N* = 46), the event commonly occurred due to vacations (58.7%), weekends (47.8%), work/school holidays (45.7%), or when medication was not needed for a period of time (39.1%). Almost 20% of patients had treatment interruption/drug holiday due to forgetting to refill medication, and 10.9% was due to management of ADHD/treatment-related complications (Fig. 1). Among patients who had a dose increase in their selected treatment regimen (*N* = 115), the increase was linked to suboptimal symptom management in 84.3% of the cases (Fig. 1). Meanwhile, the most common reason for a dose decrease (*N* = 22) was the occurrence of ADHD/treatment-related complications (45.5%; Fig. 1), mainly anxiety/panic attacks (30.0%) and insomnia and other sleep disturbances (20.0%).

The reasons for treatment changes and modifications were also explored among patients receiving different classes of stimulants and nonstimulants. Despite the smaller sample sizes of these subgroups, the main reasons underlying treatment changes and modifications were similar to that in the overall population.

### Rates of complications overall

**Fig. 2:**
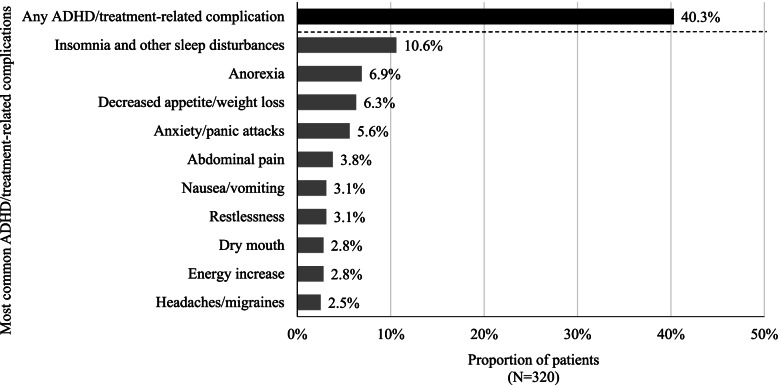
Most common ADHD/treatment-related complications. [1] Other ADHD/treatment-related complications included constipation, hypertension/increased blood pressure, tachycardia, fatigue/somnolence, heartburn, hypotension, nightmare, sweats, tremor, loss of taste, depression, obsessive compulsive disorder, postural dizziness, accidental injury, addiction to prescribed ADHD medication, autism, blurred vision, confusion/disorientation, erectile dysfunction/ejaculation dysfunction, learning disability, rash, substance abuse (other than prescribed ADHD medication), urinary issues, and other complications

The rate of ADHD/treatment-related complications documented in patients’ medical chart was assessed in the overall sample, irrespective of whether they lead to a treatment change. The results showed that 40.3% of patients had at least 1 documented ADHD/treatment-related complication (Fig. 2). Similar results were observed among patients treated with stimulants and nonstimulants (35%–43%).

**Fig. 3:**
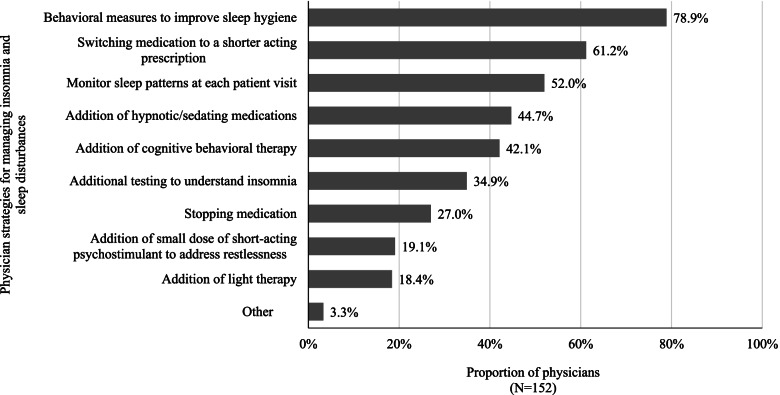
Physician strategies for managing insomnia and sleep disturbances

The most common ADHD/treatment-related complications were insomnia and other sleep disturbances (Fig. 2). Of the 152 surveyed physicians, the most frequently reported strategies for mitigating insomnia and sleep disturbances in adult patients with ADHD were behavioral interventions (78.9%), followed by treatment switching (61.2%), and monitoring of sleep patterns at each patient visit (52.0%). About 45% of physicians indicated that they had added a hypnotic/sedating medication to improve sleep for adult patients with ADHD (Fig. 3).

### Physician satisfaction and preferences regarding current treatment options

**Fig. 4:**
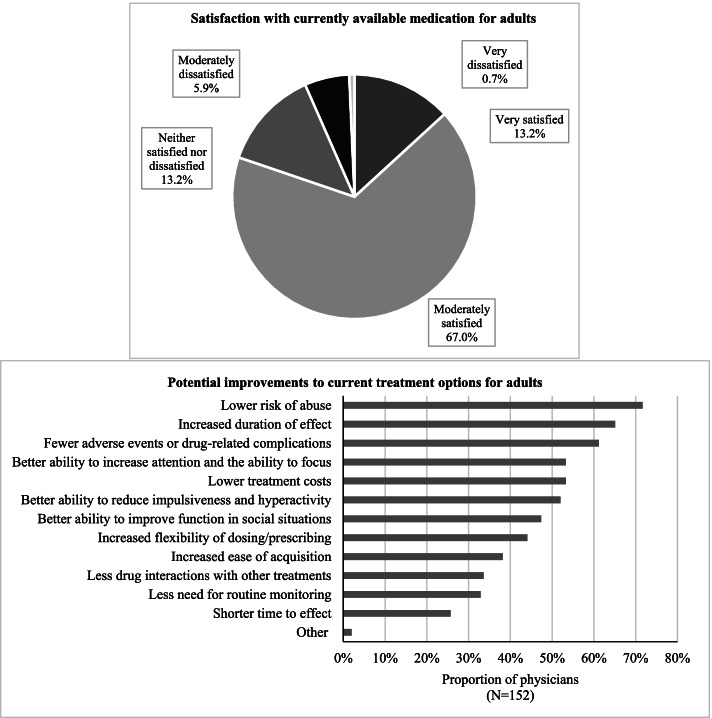
Current satisfaction with and suggested improvements to current treatment options

A survey of physicians’ level of satisfaction with available treatments for ADHD in adults and potential areas for improvement in this regard revealed that 19.8% were very dissatisfied, moderately dissatisfied, or neither satisfied nor dissatisfied with current therapeutic options (Fig. 4). The main improvements suggested by physicians were lower risk of abuse (71.7%), longer duration of effect (65.1%), and fewer ADHD/treatment-related complications (61.2%).

## Discussion

The current chart review study among adults with ADHD in the real-world setting found that the key reasons underlying treatment changes and modifications were inadequate/suboptimal symptom management and occurrence of ADHD/treatment-related complications. Additionally, over 40% of adults treated for ADHD experienced at least one complication, irrespective of whether they lead to a treatment change. Despite the availability of several therapeutic options [7, 22], our findings suggest that many outstanding challenges remain in the clinical management of the adult ADHD population. While most existing ADHD literature focused on children and adolescents, growing evidence suggests that adults with ADHD could represent a unique population that deserves more attention. For instance, diagnosing ADHD in adults could be challenging and among those who are diagnosed and treated, adherence is generally low [23, 24] and treatment changes are frequent [26]. Specifically, many adults may remain undiagnosed and untreated because of the lack of sensitivity of the Diagnostic and Statistical Manual of Mental Disorders, 5th edition (DSM-5) criteria for adults [30], a lack of clear guidelines [31], and limited awareness among physicians and patients of the condition in this population [1, 2, 24]. In fact, DSM-5 only expanded the description of ADHD presentation to include adults in 2013 [23, 24]. Even among patients who are diagnosed and treated, studies have demonstrated frequent changes to treatment regimens [25, 26, 28, 32]. In a recent claims-based study of 122,881 adult patients with ADHD, treatment changes were found to be common and occurred in about 50% of patients during the 12-month study period [26]. In addition to the relatively high incidence of discontinuation (22.5%) and switching to other ADHD drugs (17.5%), the investigators also found that treatment changes were costly and largely driven by outpatient visits [26].

While previous studies highlighted the existence of unmet treatment needs among adults with ADHD, the reasons underlying the observed treatment changes, which are essential to inform future clinical decision-making, were not captured in previous claims-based studies as claims data typically do not include detailed clinical information. Additionally, treatment modifications, such as dose changes and treatment interruptions/drug holidays, could be difficult to assess using claims data. As treatment changes and modifications may be associated with many different factors (e.g., treatment-related or patient-related), it would not be feasible to fully understand if these changes are truly associated with unmet needs without the knowledge on the underlying reasons. Therefore, the current study contributes to existing literature by examining the reasons associated with different types of treatment changes and modifications, thereby providing a more comprehensive understanding on the real-world unmet needs among adults treated with ADHD.

In regard to the reasons underlying treatment changes, the current study found that suboptimal symptom management (i.e., lack of efficacy), presence of ADHD/treatment-related complications (i.e., tolerability issue), and patient attitude/dislike of medication were among the most common reasons across the different types of treatment changes. Specifically, ADHD/treatment-related complications (mostly insomnia and other sleep disturbances) were a common reason for treatment interruption, dose decrease, and discontinuation in our study and were experienced by 40.3% of patients while they were on the selected treatment regimen. This proportion of patients included those who experienced ADHD/treatment-related complications but did not have a treatment change, implying that the true disease burden experienced by adults with ADHD is likely beyond that could be observed among patients with documented treatment changes. For instance, the current physician survey found that the management of insomnia and other sleep disturbances involved additional interventions, monitoring, and medications (e.g., hypnotics/sedatives). These additional measures and medications not only add to the burden of both physicians and patients, but some of these medications may also be associated with additional complications [33, 34]. It should also be noted that the proportion of patients with documented ADHD/treatment-related complications in this study (i.e., 40.3%) only included patients who experienced complications *and* decided to seek medical services to discuss the issues with the physicians. Thus, this proportion may even be higher in the real world considering not all patients would seek help or report all complications to their physicians. Meanwhile, the reasons for treatment changes due to patient attitude/dislike of medication could be multifactorial and related to a combination of lack of effectiveness, treatment complications, dosing inconvenience, stigma, or other patient factors that led the physician to consider that the patient disliked the medication. It is notable that these factors contributing to patients’ dislike of the medication may not be mutually exclusive from other reasons for treatment changes; this suggests that addressing other medication-related unmet needs may also potentially improve patient’s overall attitude toward the medication. Lastly, reasons for remaining on the same treatment regimen despite experiencing complications may include the consideration that managing the complications could be less complex than altering the therapeutic strategy. It may also be possible that some patients may have cycled through multiple treatment options and lack better alternatives. Collectively, our results imply that more effective and better tolerated therapies are needed for adult patients with ADHD.

The results of the physician survey conducted in the current study showed that about 20% of surveyed physicians reported being very dissatisfied, moderately dissatisfied, or neither satisfied nor dissatisfied with current ADHD treatment options for adults. While one might expect a higher degree of dissatisfaction among physicians given the previously reported frequent treatment changes in adult patients with ADHD [26], future studies should also assess patient satisfaction to fully apprehend the patients’ perspectives on treatment needs. The results of these patient studies may serve as meaningful references for future treatment development, which should take into account areas of unmet needs considered most important by the patients. Notably, potential improvements to current treatment options cited by physicians (i.e., a lower risk of abuse, increased duration of effect, and fewer adverse events or drug-related complications) aligned with the common reasons for treatment changes (i.e., ADHD/treatment-related complications and suboptimal management of symptoms). This finding further supports the potential benefit of addressing the unmet need in the current ADHD treatment landscape.

The current study provides important context around the issue of frequent treatment changes among adult patients with ADHD by examining the underlying reasons for the changes, which may help physicians engage in specific discussions with patients and families and/or prompt them to monitor specific events and symptoms more closely. For instance, by learning that almost one-fifth of patients had treatment interruption/drug holiday due to forgetting to refill medication, physicians may educate patients and families on the importance of adherence and suggest steps to ensure patients remember to refill medications on time (e.g., family members may play a greater role in providing reminders, or patients may install reminder applications on their mobile phones), which may promote treatment continuity, and in turn improve treatment outcome. Additionally, frequent treatment changes have recently been shown to be associated with excess healthcare costs in adults with ADHD [26]. Thus, increasing physician and patient awareness around the frequency and consequences of ADHD/treatment-related complications in adults may guide physicians during treatment decision-making and help patients/families make more informed choices of the treatments. Notably, anxiety/panic attacks were the most common complications leading to discontinuation. Given that patients with ADHD frequently have comorbid mood and anxiety disorders [8–11], these complications should be factored into treatment decision-making and patient management. For example, physicians can be more vigilant in assessing mood change and anxiety symptoms in their ADHD patients and consider alternate medications or treatment strategies that may exert less adverse effects in these regards for at-risk patients. Future studies are also warranted to assess how certain complications such as anxiety may affect treatment patterns in ADHD. The current results also point to the importance of developing new therapies with improved tolerability and efficacy profiles. Stimulants remain the primary treatment options for most adult patients with ADHD because of its efficacy in reducing core symptoms [7]; however, many patients treated with stimulants experience insomnia and other sleep disturbances, as reflected in the current study. Physicians often tended to manage these complications through adding hypnotic/sedating medication or recommending behavioral therapies, which highlights the potential lack of alternative options in ADHD management and the need to develop efficacious medications with less impact on patients’ sleep quality. Based on the results of the current study, improved medications may also help alleviate the problems of frequent treatment changes and the undesired clinical and economic consequences.

This study had some limitations. First, data captured by the study’s online survey and reporting form were limited to information available in patients’ medical records kept by the physicians who participated in the study; information on healthcare services received outside the physician’s care setting that were not recorded in the medical chart may not have been available. Second, although participating physicians were instructed to select patients at random, there may have been selection bias—for example, for patients who were recently seen by the treating physician or with favorable/unfavorable outcomes. Additionally, the effects of any inaccuracies in reporting by physicians on the study’s findings are unknown. Third, it should be noted that a potential lack of efficacy may be improved by dosing changes, or other physician actions, and may not always be inferred as dissatisfaction toward the medication. Fourth, the rate of some complications contributing to a treatment change may not be accurately captured due to small sample size and low prevalence of some complications. Fifth, the current survey did not differentiate whether the ADHD/treatment complications were adverse event–related or comorbidity-related. Finally, although the study included data from a diverse set of physicians and patients, the results may not be generalizable to the entire ADHD physician and patient populations in the US such as physicians with different specialties and practice types, patients with different treatment history, or older patients with ADHD.

## Conclusions

The results of this chart review study—considered together with previous treatment pattern work in adult ADHD—suggest that despite many ADHD drug products available, a large unmet need still exists. Specifically, lack of efficacy and ADHD/treatment-related complications are the most common reasons leading to a treatment change. The rate of complications, irrespective of whether they lead to a treatment change, is also high, with insomnia and sleep disturbances being the most common complications overall. Our findings highlight the importance of developing more effective and safer treatments to alleviate the individual and societal burden of adult ADHD.

## Data Availability

The data analyzed in this study are subject to Health Insurance Portability and Accountability Act privacy restrictions and are not publicly available. De-identified data could be made available by the corresponding author upon reasonable request.

## Abbreviations

ADHD: Attention-deficit/hyperactivity disorder
DSM-5: Diagnostic and Statistical Manual of Mental Disorders, 5th edition
